# The caligid life cycle: new evidence from *Lepeophtheirus elegans* reconciles the cycles of *Caligus* and *Lepeophtheirus* (Copepoda: Caligidae)

**DOI:** 10.1051/parasite/2013015

**Published:** 2013-05-07

**Authors:** Balu Alagar Venmathi Maran, Seong Yong Moon, Susumu Ohtsuka, Sung-Yong Oh, Ho Young Soh, Jung-Goo Myoung, Anna Iglikowska, Geoffrey Allan Boxshall

**Affiliations:** 1 Marine Ecosystem Research Division, Korea Institute of Ocean Science & Technology P.O. Box 29 Seoul 425-600 Korea; 2 Faculty of Marine Technology, Chonnam National University Yeosu 550-749 Korea; 3 Takehara Marine Science Station, Setouchi Field Science Centre, Graduate School of Biosphere Science, Hiroshima University 5-8-1 Minato-machi Takehara, Hiroshima 725-0024 Japan; 4 Marine Ecology Department, Institute of Oceanology Polish Academy of Sciences Powstańców Warszawy 55 81-712 Sopot Poland; 5 Department of Life Sciences, Natural History Museum Cromwell Road London SW7 5BD UK

**Keywords:** Caligidae, Copepoda, sea louse, life cycle, larval stages, developmental stages

## Abstract

The developmental stages of the sea louse *Lepeophtheirus elegans* (Copepoda: Caligidae) are described from material collected from marine ranched Korean rockfish, *Sebastes schlegelii*. In *L. elegans*, setal number on the proximal segment of the antennule increases from 3 in the copepodid to 27 in the adult. Using the number of setae as a stage marker supports the inference that the post-naupliar phase of the life cycle comprises six stages: copepodid, chalimus I, chalimus II, pre-adult I, pre-adult II, and the adult. We observed variation in body length in both of the chalimus stages which we consider represents an early expression of sexual size dimorphism. We interpret the larger specimens of chalimus I as putative females, and the smaller as putative males; similarly with chalimus II, larger specimens are putative females and the smaller are males. Two patterns of life cycle are currently recognized within the Caligidae but the evidence presented here reconciles the two. We conclude that the typical caligid life cycle comprises only eight stages: two naupliar, one copepodid, and four chalimus stages preceding the adult in *Caligus*, but with the four chalimus stages represented by two chalimus and two pre-adult stages in *Lepeophtheirus*. This is a profound change with significant implications for the aquaculture industry, given that lice monitoring protocols include counts of chalimus stages and use temperature to predict when they will moult into the more pathogenic, mobile pre-adults. Lice management strategies must be tailored to the precise life cycle of the parasite.

## Abstract (fr)

Les stades de développement du copépode parasite *Lepeophtheirus elegans* (Copepoda : Caligidae) sont décrits à partir de matériel collecté sur des spécimens du Sébaste coréen *Sebastes schlegelii* élevés en mer. Chez *L. elegans,* le nombre de soies sur le segment proximal de l’antennule croît de trois au stade copépoditique à 27 chez l’adulte. L’utilisation du nombre de soies comme marqueur du stade permet d’établir que la phase post-nauplienne du cycle de vie comprend six stades: copépodite, chalimus I, chalimus II, pré-adulte I, pré-adulte II et adulte. Nous avons observé une variation de la longueur du corps chez les deux stades chalimus qui représentent, à notre avis, une expression précoce du dimorphisme sexuel concernant la taille. Nous interprétons les plus grands spécimens de chalimus I comme de futures femelles, et les plus petits comme de futurs mâles; de même, pour les chalimus II, les plus grands spécimens sont femelles et les plus petits sont mâles. Deux modèles de cycles de vie sont actuellement reconnus chez les Caligidae, mais les observations présentées ici réconcilient les deux. Nous concluons que le cycle de vie typique des Caligidae comprend seulement huit stades : deux naupliens, un copépodite et quatre stades chalimus précédant l’adulte chez *Caligus*, mais avec les quatre stades chalimus représentés par deux stades chalimus et deux stades pré-adultes chez *Lepeophtheirus*. Ceci est un changement profond avec des implications significatives pour l’industrie de l’aquaculture, puisque la surveillance des poux de mer implique des comptages des stades chalimus et utilise la température pour prédire quand ils vont muer en pré-adultes mobiles, qui sont plus pathogènes. Les stratégies de contrôle des copépodes parasites doivent être adaptées au cycle de vie exact du parasite.

## Introduction

Even though the family Caligidae currently comprises in excess of 450 valid species [5, 6, 8], information on the complete life cycle is available for just 17 species. These species belong to just three genera: *Caligus* Müller, 1785 [33] (12 species), *Lepeophtheirus* von Nordmann, 1832 [44] (4 species), and *Pseudocaligus* A. Scott, 1901 [40] (1 species) [16, 30, 35]. In all caligids thus far studied the eggs are carried in linear, uniseriate egg strings and hatch as a free-swimming nauplius. The free-swimming phase consists of two naupliar stages followed by the copepodid, which is the infective stage and locates the host. Subsequent development on the host appeared to be more variable, with different numbers of stages having been reported in different species of *Caligus* and *Lepeophtheirus*: four or six chalimi (chalimus stages), zero or two pre-adults, and one adult stage (see Discussion in Ref. [16]). All copepods for which the life cycle is known have a maximum of six stages during the post-naupliar phase, with a single exception, *Lepeophtheirus*, which had eight post-naupliar stages according to published reports [4, 19, 28, 45]. However, when Ohtsuka *et al*. [35] elucidated the full life cycle of *Pseudocaligus fugu* Yamaguti, 1936 [48] on tiger puffer, *Takifugu rubripes* (Temminck and Schlegel, 1850), they postulated that the life cycle in *Lepeophtheirus* species may have been misinterpreted. They considered that, in existing descriptions of the developmental stages of *Lepeophtheirus* species, the differences between chalimus I and II and between chalimus III and IV might be explained only by intramoult growth and development. They recommended that this novel interpretation should be tested.

Worldwide, 122 described species of *Lepeophtheirus* are accepted as valid [46] and of these, the life cycle of four species has been revealed, namely, *L. dissimulatus* Wilson, 1905 [47] [28], *L. hospitalis* Fraser, 1920 [11] [45], *L. pectoralis* (Müller, 1776) [32] [4], and *L. salmonis* (Krøyer, 1837) [26] [19]. In this study, the complete life cycle of a fifth species, *Lepeophtheirus elegans* Gusev, 1951 [13], is described.

*Lepeophtheirus elegans* has so far been reported from four fish hosts, two stichaeids, *Chirolophis japonicus* Herzenstein (= *Azumma japonica, A. emmnion*) and *Pholidapus dybowskii* (Steindachner) from Russia [13], Japan [15, 42], and Korea [23, 25], and a pholid, *Pholis picta* (Kner), and cottid, *Myoxocephalus brandtii* (Steindachner), both from Russian waters [13]. Here we report this copepod from the Korean rockfish, *Sebastes schlegelii* Hilgendorf, which is the subject of marine ranching at Tongyeong marine living resources research and conservation center (TMRC) in Korea. Since *L. elegans* has now been reported from hosts representing four families (Stichaeidae, Pholidae, Cottidae, and Sebastidae), it could be considered to exhibit relatively low host specificity. The Korean rockfish is widely distributed in the coastal waters of the northwest Pacific [12] and has become a valuable aquaculture species, replacing Olive flounder as the most commonly cultured species in Korea, even though it was well behind the olive flounder in culture until 1995 [23]. Recently, Venmathi Maran *et al.* [43] reported another sea louse, *Caligus sclerotinosus* Roubal, Armitage et Rohde, 1983 [38], as an aquaculture pest in Korea. *Lepeophtheirus elegans* should also be considered as a pest due to the severe infection on rockfish found along the southern coast of Korea. During a survey conducted from June 2011 to February 2012 at the TMRC 45 ranched Korean rockfish were collected (five per month; ranging from 10 to 26 cm in total length). They were severely infected by *L. elegans* on the body surface and fins. The prevalence was 98.8% and the maximum number of individuals per host was 29, with a mean intensity of 7.24. We need to better understand the biology of this pest species in order to develop effective management and control strategies for infections in commercial aquaculture facilities. Here we describe all the developmental stages of *L. elegans* and compare its life cycle with that of other sea lice.

## Materials and methods

Ovigerous females of *L. elegans* were collected from the body surface of marine ranched *Sebastes schlegelii* (Sebastidae) at TMRC, Tongyeong, Gyeongsangnam-do, Korea in September 2011. Egg strings from these females were incubated at a temperature of ca. 17–20 °C in sterilized seawater until hatching, and then through to the appearance of the copepodid stage. Chalimus stages, pre-adults, and adults were collected from the fins and body surface of the same host species, collected at TMRC from June to December 2011. The copepods were cleared in 85% lactic acid for about 4 h, dissected, and examined following the wooden slide procedure of Humes & Gooding [17]. Drawings and measurements were made with the aid of a drawing tube attached to an Olympus BX51 differential interference contrast microscope. Specimens were measured intact using an ocular micrometer and measurements are given as means followed by the range in parentheses. Anatomical terminology follows Huys & Boxshall [18] and fish names conform to FishBase [12]. The term pre-adult is used for a life cycle stage which is mobile over the host body surface immediately after moulting from the preceding stage and attaches by a frontal filament prior to moulting to the next stage. In contrast, a chalimus stage is attached by a frontal filament for the duration of the stage. Spines and setae are not distinguished in the setal formula given for chalimus I and II. In the copepodid, pre-adult and adult stages spines are given by Roman numerals and setae by Arabic numerals.

## Results

### The developmental stages of *Lepeophtheirus elegans* Gusev, 1951

#### First nauplius (Figure 1A–E)

**Figure 1.**
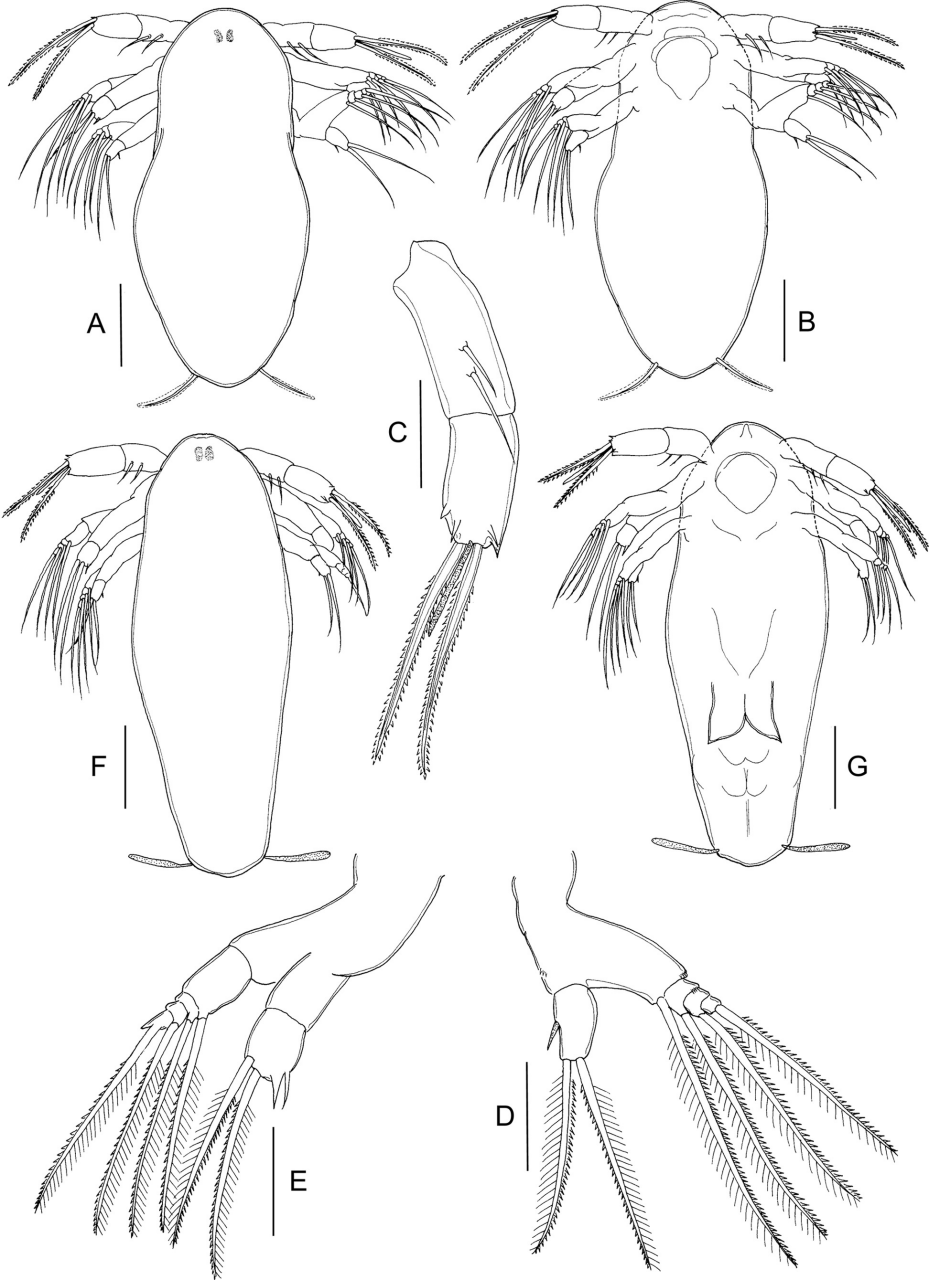
*Lepeophtheirus elegans* Gusev, 1951. First nauplius (A–E): A, habitus, dorsal; B, habitus, ventral; C, antennule; D, antenna; E, mandible. Second nauplius (F, G): F, habitus, dorsal; G, habitus, ventral. Scale bars: A, B, F, G = 0.1 mm; C–E = 0.05 mm.

Body (Figure 1A, B) ovoid, widest at midlength. Nauplius eye present. Labrum produced anteroventrally, mouth not open. Paired balancers located posterolaterally, curved outwards. Body length: mean 0.49 mm (range 0.46–0.52 mm) (*n* = 5).

Antennule (Figure 1C) two-segmented; proximal segment longer, with two marginal setae; distal segment separated from proximal by distinct articulation; distal segment with four short spiniform elements subterminally around apex, plus two serrate setae and one short aesthetasc terminally. Antenna (Figure 1D) biramous, with protopod indistinctly subdivided into coxa and basis; basis not separated from proximal segments of rami. Exopod four-segmented; all four segments each bearing inner distal seta. Endopod two-segmented; free second segment armed with one short medial seta and two long setae terminally. Long setae on both rami ornamented with serrated outer and plumose inner margins. Mandible (Figure 1E) biramous, with unsegmented protopod not separated from proximalmost exopodal segment; free second to fifth exopodal segments each bearing long seta similar to those on antenna. Endopod with single free segment bearing two long terminal setae similar to those on antenna, and two short naked setae located distally on inner margin.

#### Second nauplius (Figures 1F, G and 2A–C)

Body (Figure 1F, G) more slender than preceding stage, with traces of developing post-mandibular limbs; nauplius eye present; balancers with proximal part narrow and distal part flattened. Body length: 0.54 mm (0.52–0.55 mm) (*n* = 9).

**Figure 2.**
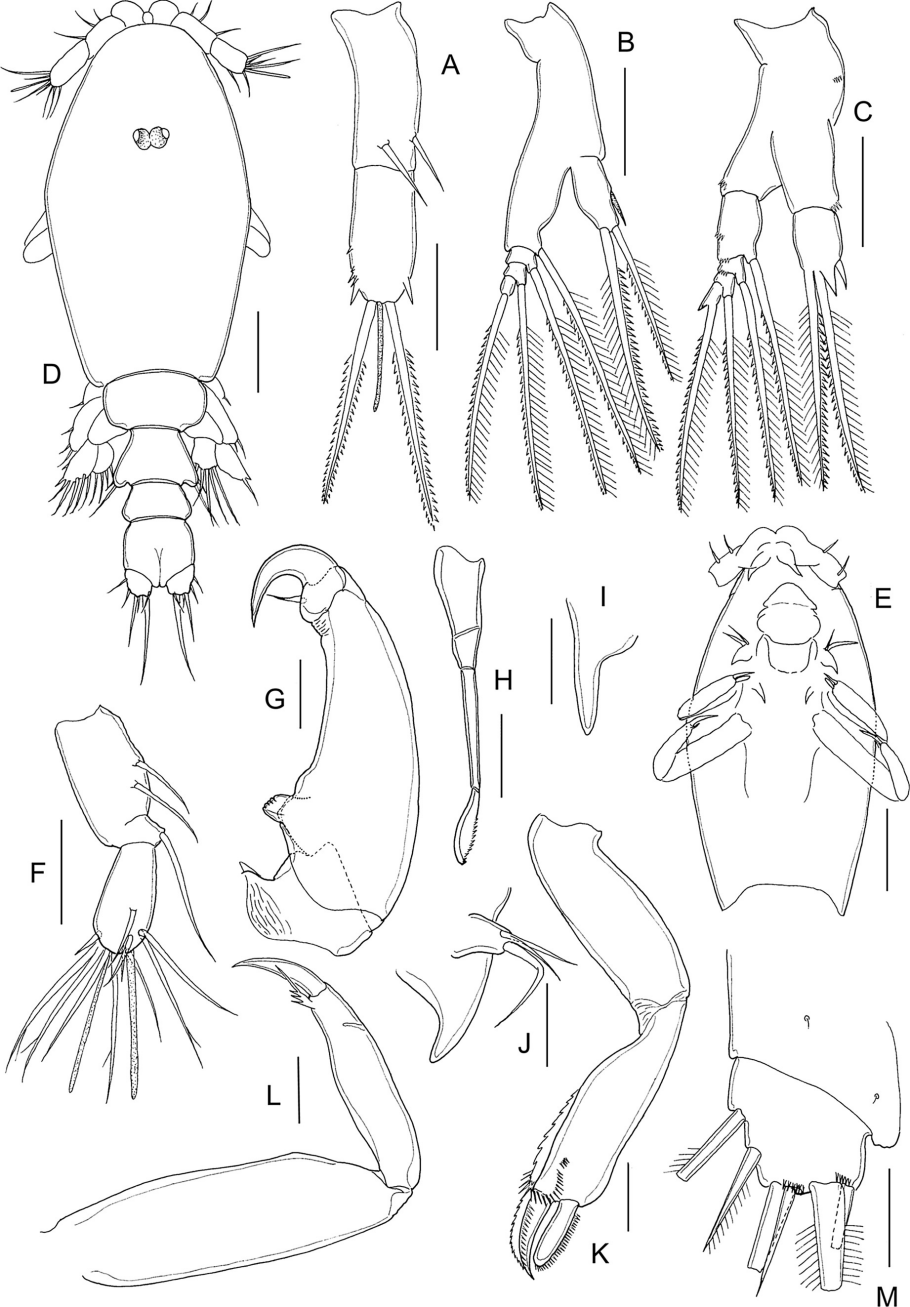
*Lepeophtheirus elegans* Gusev, 1951. Second nauplius (A–C): A, antennule; B, antenna; C, mandible. Copepodid (D–M): D, habitus, dorsal; E, cephalothorax, ventral; F, antennule; G, antenna; H, mandible; I, post-oral process; J, maxillule; K, maxilla; L, maxilliped; M, caudal ramus, ventral. Scale bars: A–C, F = 0.05 mm; D, E = 0.01 mm; G–M = 0.025 mm.

Antennule (Figure 2A) as in preceding stage except for three additional rudimentary setae on distal segment. Antenna (Figure 2B) and mandible (Figure 2C) as in preceding stage. Bifid structure comprising paired, posteriorly directed processes, present on ventral midline (Figure 1G).

#### Copepodid (Figures 2D–M and 3)

**Figure 3.**
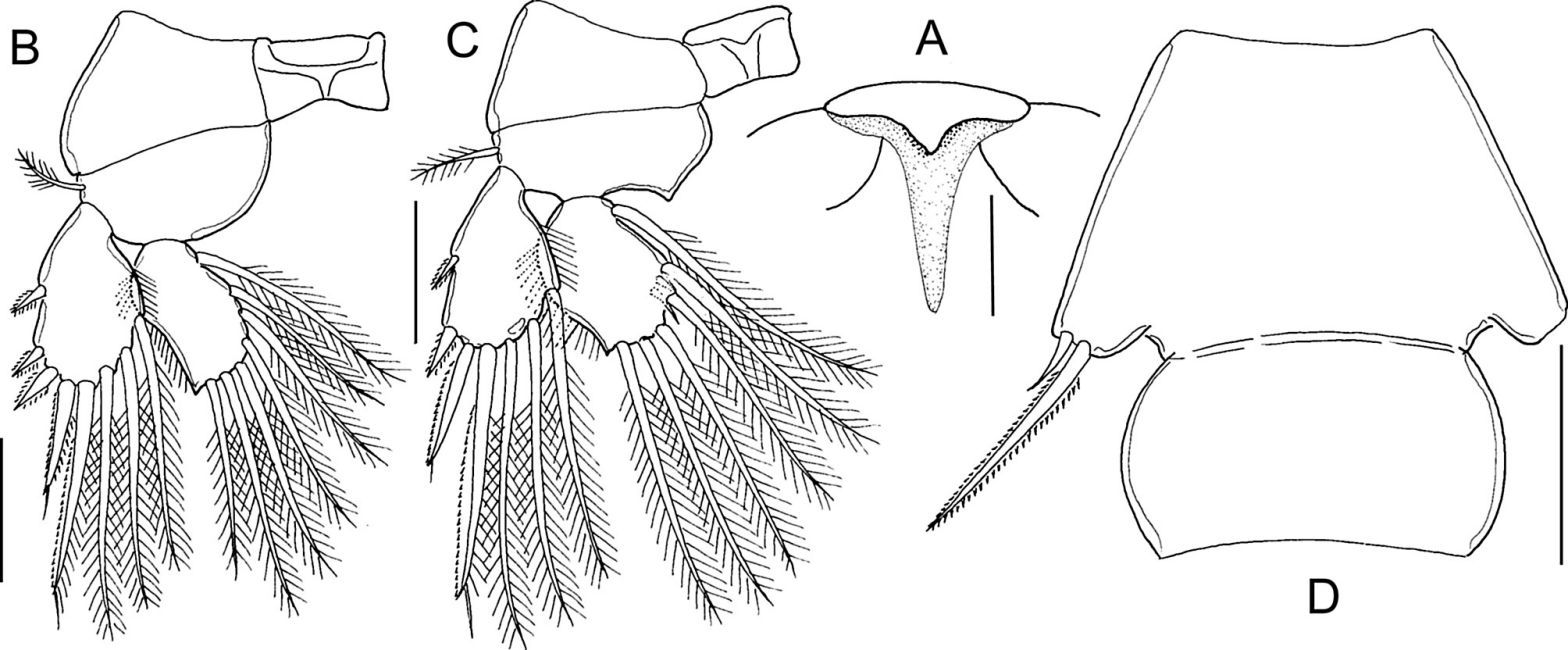
*Lepeophtheirus elegans* Gusev, 1951. Copepodid: A, rostrum, ventral; B, leg 1; C, leg 2; D, third pedigerous somite and leg 3. All scales = 0.05 mm.

Body (Figure 2D–E) with dorsal surface highly pigmented from anterior part of cephalothorax to caudal rami (pigmentation not illustrated). Cephalothorax incorporating first pedigerous somite, about 1.5 times longer than free post-cephalothoracic somites and caudal rami combined; widest about at midlength. Rostrum (Figure 3A) weakly developed, with conical posteriorly directed process. Second pedigerous somite free, wider than long; third pedigerous somite with paired anlagen of leg 3 (Figures 3D and 2D); third free somite slightly smaller than preceding somite, unarmed; fourth somite (anal somite) with short caudal rami (Figure 2M) bearing six setae. Body length: 0.72 mm (0.70–0.74 mm) (*n* = 6).

Antennule (Figure 2F) with distinctly but incompletely subdivided proximal segment bearing three long setae anteroventrally; distal segment bearing 2 aesthetascs and 11 setae, 4 of which with bifid tip. Antenna (Figure 2G) three-segmented; first segment small, unarmed; second segment largest, with conspicuous rugose process at proximal one-third of inner margin; third segment armed with minute inner seta proximally and bearing smoothly recurved claw. Mandible (Figure 2H) stylet-like, consisting of four parts; third part longest; distal part bearing about 10 teeth along inner margin. Maxillule (Figure 2J) comprising weakly curved posterior process plus anterior papilla armed with three unequal setae. Pair of short, pointed post-oral processes (Figure 2I) located between maxillule and maxilliped. Maxilla (Figure 2K) two-segmented; first segment unarmed; second segment as long as first, with calamus about as long as canna, and with flabellum located distal to midpoint of outer margin. Maxilliped (Figure 2L) subchelate; proximal protopodal segment just longer than distal subchela; subchela comprising unarmed first endopodal segment and distal segment separated by partial suture, carrying terminal claw and trifid setal element.

Legs 1 (Figure 3B) and 2 (Figure 3C) biramous with distinct, one-segmented rami; protopods divided into coxa and basis; intercoxal sclerite present. Inner seta on basis of leg 1 absent. Seta and spine formula is given as follows:

**Table:** 

	Coxa	Basis	Exopod	Endopod
Leg 1	0–0	1–0	III, I, 4	7
Leg 2	0–0	1–0	II, I, 4	6

Leg 3 (Figure 3D) represented by short posterolateral process bearing one short spine and one serrate seta.

#### First chalimus (Figures 4 and 5)

**Figure 4.**
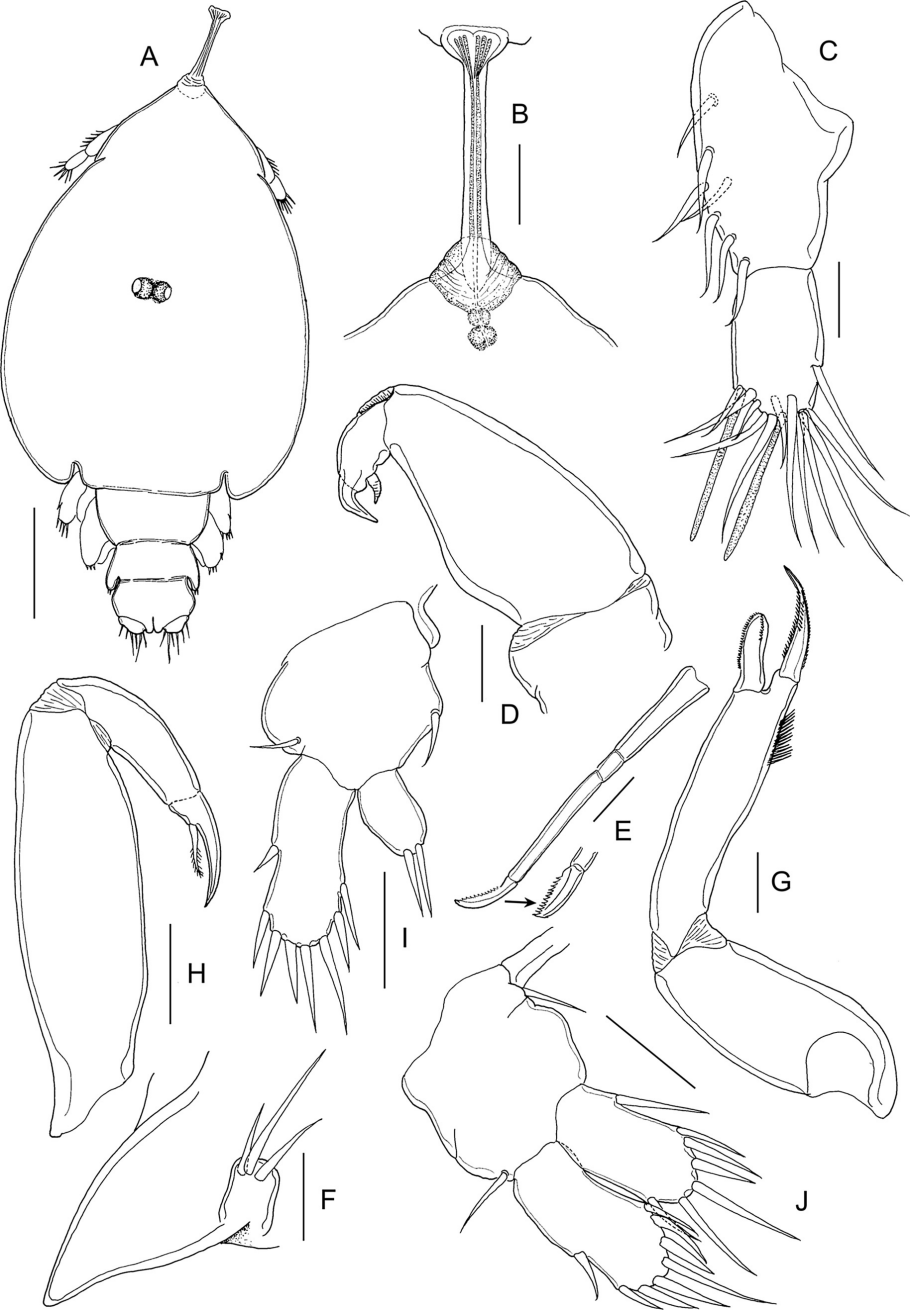
*Lepeophtheirus elegans* Gusev, 1951. First chalimus: A, habitus, dorsal; B, frontal filament; C, antennule; D, antenna; E, mandible; F, maxillule; G, maxilla; H, maxilliped; I, leg 1; J, leg 2. Scale bars: A = 0.2 mm; B = 0.05 mm; C–J = 0.025 mm.

**Figure 5.**
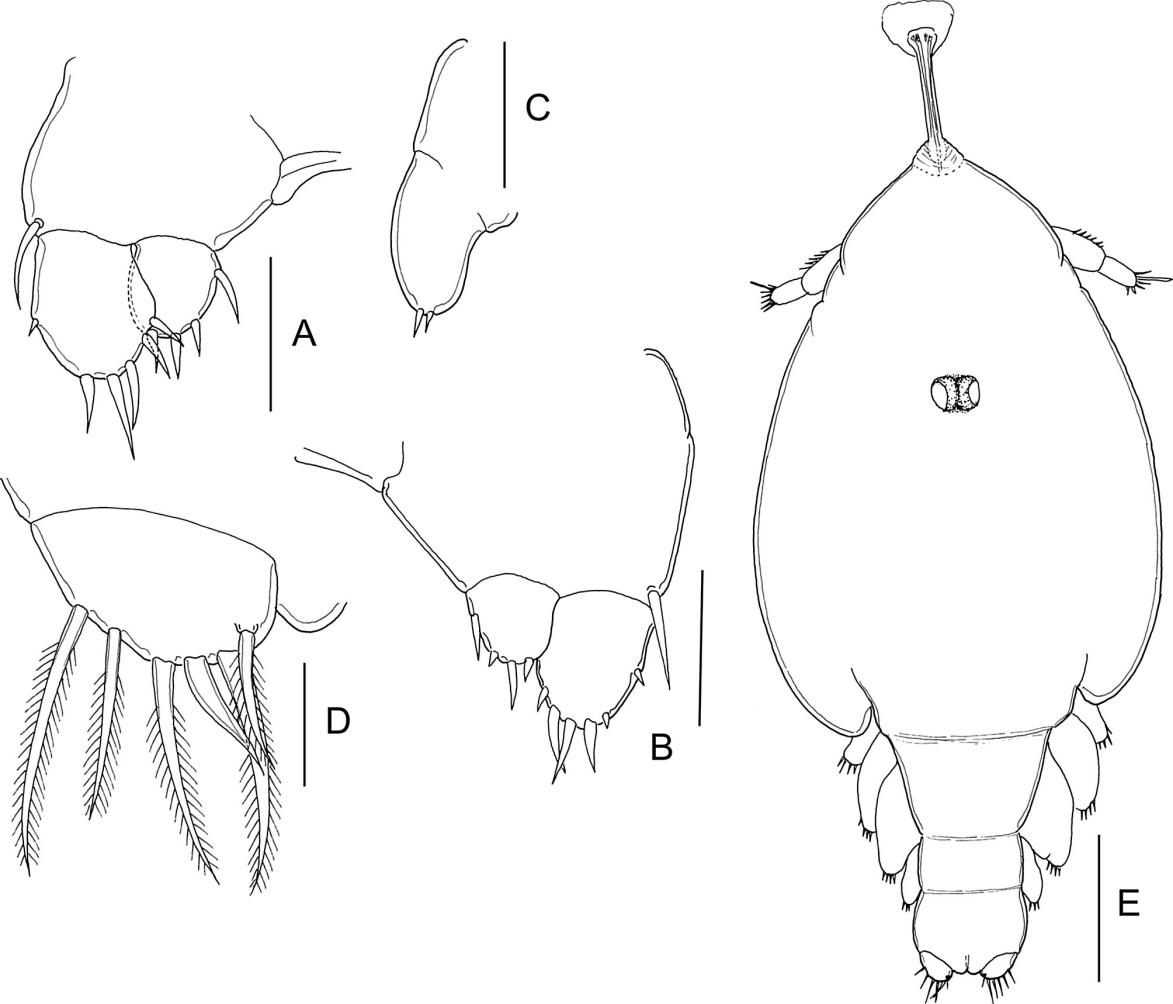
*Lepeophtheirus elegans* Gusev, 1951. First chalimus: A, leg 3; B, leg 3 (other specimen); C, leg 4; D, caudal ramus; E, habitus of putative female, dorsal. Scale bars: A–D = 0.025 mm; E = 0.2 mm.

Body (Figures 4A and 5E) attached to host by short frontal filament (Figure 4B). Cephalothorax about 2.5 to 3 times longer than free posterior somites combined. Frontal margin protruded anteriorly to form triangular plate. First free (= second pedigerous) somite about 1.5 times to 2.0 wider than long (Figure 5E); second free (= third pedigerous) somite narrower than first; anal somite bearing small caudal rami (Figure 5D) armed with six setae of unequal length. Body length: putative male 1.08 mm (1.07–1.09 mm) (*n* = 5) (Figure 4A): putative female 1.18 mm (1.12–1.21 mm) (*n* = 5) (Figure 5E).

Antennule (Figure 4C) two-segmented; proximal segment bearing 7 setae; distal segment with 12 setae and 2 aesthetascs. Antenna (Figure 4D) modified from that of preceding copepodid stage; consisting of broad basal segment; middle segment with smoothly convex medial margin; distal segment with curved distal claw bearing short inner setal element. Mandible (Figure 4E) stylet-like structure with 12 marginal teeth subterminally, as in adult (cf. Figure 12D). Maxillule (Figure 4F) with posterior process broad and pointed, papilla with three unequal setae. Maxilla (Figure 4G) two-segmented, first segment unarmed, second segment longer than first, with broad calamus and slightly longer canna ornamented with minutely serrated membrane, and with hairy flabellum located distally on outer margin. Maxilliped (Figure 4H) indistinctly three-segmented, protopodal segment robust; distal endopodal segment of subchela bearing curved claw and short inner seta. Sternal furca absent.

Leg 1 (Figure 4I) biramous with unequal, one-segmented rami; exopod elongate, bearing eight naked setal elements; endopod reduced from copepodid, comprising short segment armed with two naked setae apically. Leg 2 (Figure 4J) with both rami more elongate than in preceding stage but both unsegmented; exopod bearing eight naked setal elements; endopod with seven naked setae. Leg 3 biramous (Figure 5A), protopod with outer seta; intercoxal plate present; exopod bearing six naked setal elements and endopod with four naked setae; size of rudimentary setae variable (cf. Figures 5A and 5B). Seta and spine formula is given as follows:

**Table:** 

	Coxa	Basis	Exopod	Endopod
Leg 1	0-0	1-1	8	2
Leg 2	0-1	1-0	8	7
Leg 3	(1-0)		6	4

Leg 4 (Figure 5C) rudimentary, lobate, with two small setal elements on apex.

#### Second chalimus (Figures 6 and 7)

**Figure 6.**
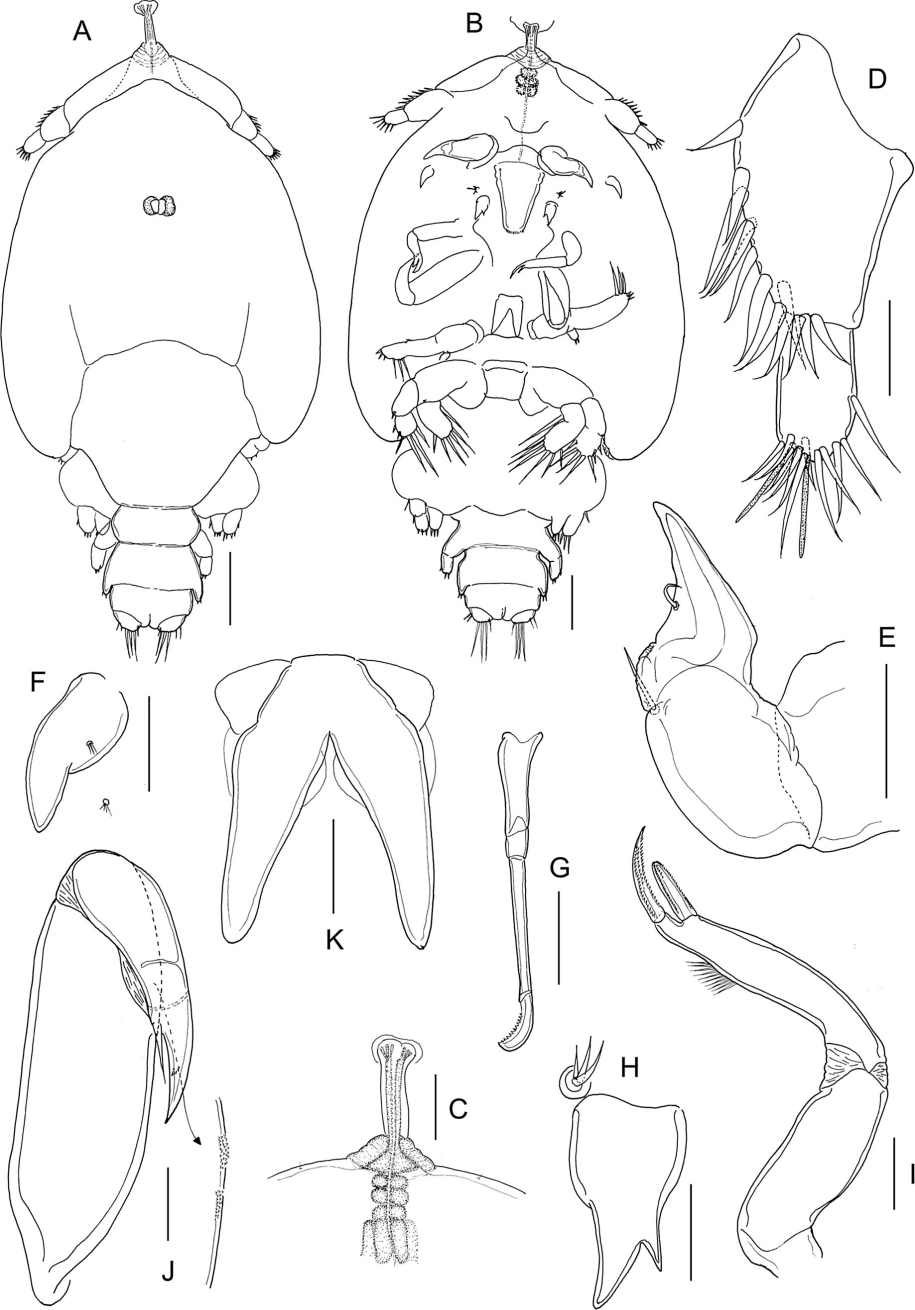
*Lepeophtheirus elegans* Gusev, 1951. Second chalimus: A, habitus, dorsal; B, habitus, ventral; C, frontal filament; D, antennule; E, antenna; F, postantennary process; G, mandible; H, maxillule; I, maxilla; J, maxilliped; K, sternal furca. Scale bars: A = 0.05 mm; B = 0.2 mm; C–K = 0.05 mm.

**Figure 7.**
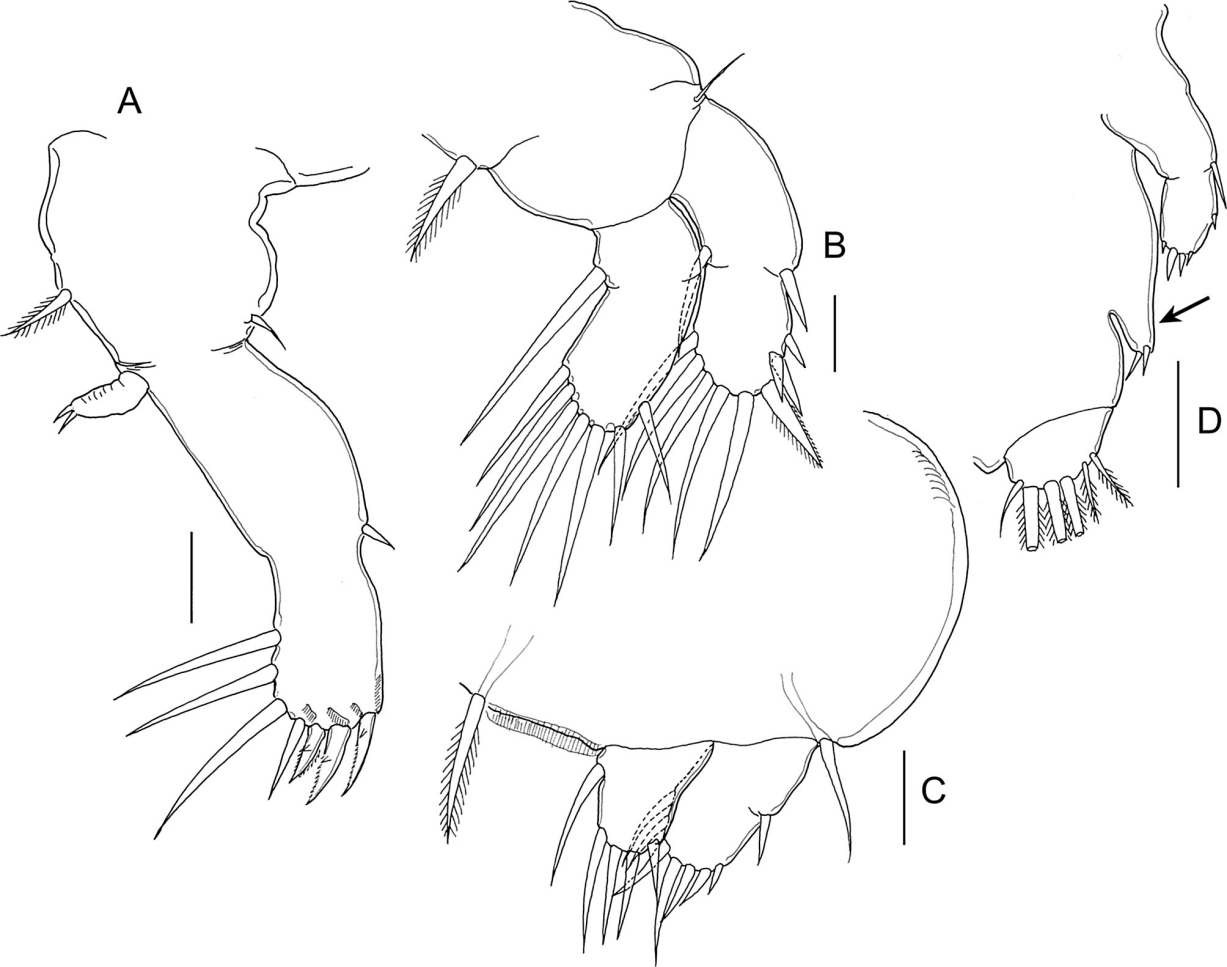
*Lepeophtheirus elegans* Gusev, 1951. Second chalimus: A, leg 1; B, leg 2; C, leg 3; D, legs 4 and 5 (arrowed). Scale bars: A–D = 0.1 mm.

Body (Figure 6A, B) with cephalothorax laterally expanded and incorporating both first and second pedigerous somites; cephalothorax about 3.5 times longer than free posterior somites combined; anterior margin with frontal filament (Figure 6C). Third pedigerous somite free. Fourth pedigerous somite bearing paired anlagen of leg 4 ventrolaterally. Caudal ramus (Figure 7D) broader than in preceding stage, with three short and three long setae. Body length: putative male 1.68 mm (1.57–1.72 mm) (*n* = 7); putative female 2.02 mm (1.89–2.32 mm) (*n* = 6).

Antennule (Figure 6D) proximal segment bearing 13 setae anteriorly; distal segment with 12 setae plus 2 aesthetascs. Antenna (Figure 6E) three-segmented, proximal segment broad; middle segment with rudiment of dorsal adhesion pad discernible; distal segment with two short setae. Postantennary process (Figure 6F) first appearing at this stage; pointed, with one multisensillate papilla on surface and one on adjacent surface of cephalothorax. Mandible (Figure 6G) unchanged. Maxillule (Figure 6H) with anterior papilla and posterior process clearly defined; posterior process bifid with small secondary inner tine present. Maxilla (Figure 6I) as in preceding stage. Maxilliped (Figure 6J) with segments comprising subchela more completely fused than in preceding stage; rugose patches of minute denticles present distally on medial surface. Sternal furca (Figure 7K) present.

Leg 1 (Figure 7A) sympod indistinctly segmented, medial seta pinnate; exopod indistinctly two-segmented, proximal segment larger, with short unarmed spine at outer distal angle; distal segment with four short elements on distal margin and three longer naked setae on inner margin; endopod and apical setae further reduced in size, vestigial.

Leg 2 (Figure 7B) sympod unsegmented, medial seta pinnate; both rami indistinctly two-segmented; proximal segment of exopod with unarmed seta at inner distal angle and robust spine at outer distal angle; distal segment with three short spines on lateral margin, longer spine at outer distal angle, and five long setae around inner and distal margins; proximal segment of endopod with one long seta on medial margin, distal segment with seven long setae around margin.

Leg 3 (Figure 7C) broad sympod unsegmented, armed with outer protopodal seta on margin lateral to base of exopod and stout pinnate seta (inner coxal seta) on posterior margin: exopod showing trace of incipient subdivision into two segments; proximal segment with one spine at outer distal angle, distal segment with seven setal elements; endopod unsegmented with one seta on medial margin and four setae distally; strip of marginal membrane present on sympod medial to endopod. Setal formula is given as follows:

**Table:** 

	Coxa	Basis	Exopod	Endopod
Leg 1	0-0	1-1	8	2
Leg 2	0-1	1-0	11	8
Leg 3	(1-1)		8	5

Leg 4 (Figure 7D) uniramous; protopod indistinctly separate from developing exopod, armed with outer distal seta; exopod with five rudimentary elements. Leg 5 (arrowed on Figure 7D) represented by lobate outgrowth bearing two rudimentary setae at apex.

#### Pre-adult I, female (Figures 8 and 9A–E)

**Figure 8.**
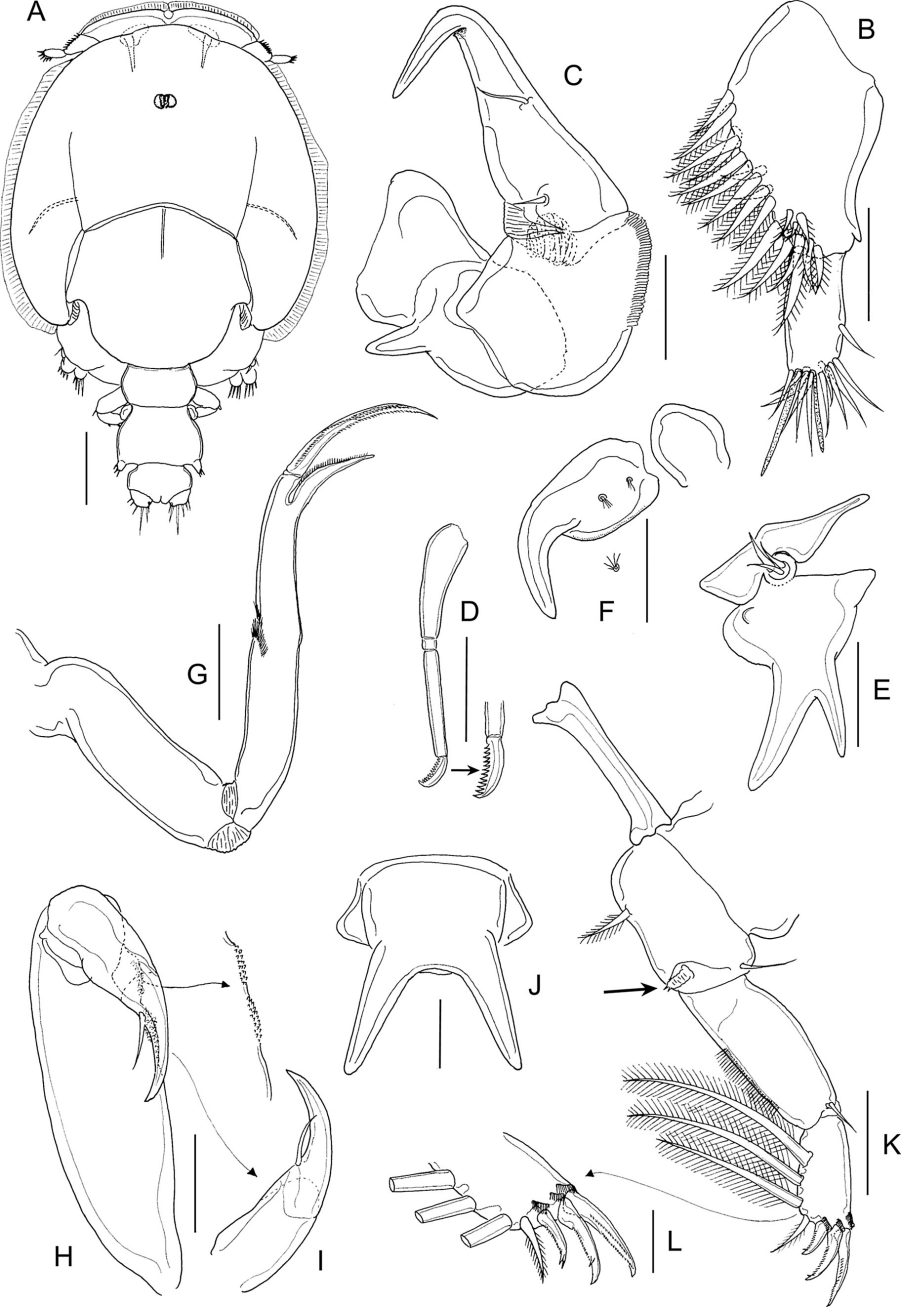
*Lepeophtheirus elegans* Gusev, 1951. Pre-adult I, female: A, habitus, dorsal; B, antennule; C, antenna: D, mandible; E, maxillule; F, postantennary process; G, maxilla; H, maxilliped; I, terminal part of maxilliped; J, sternal furca; K, leg 1 (with vestigial endopod arrowed); L, tip of leg 1 exopod. Scale bars: A = 0.4 mm; B–H, K = 0.1 mm; J, L = 0.05 mm.

Body (Figure 8A) with fully developed cephalothorax of adult caligid form, incorporating third pedigerous somite and with well-developed frontal plates. Frontal plates, lateral margins of cephalothorax, and posterior sinuses all with marginal membrane. Typical H-shape suture line markings visible on dorsal side of cephalothoracic shield. Fourth pedigerous somite free, wider than long. Genital complex wider than long, with parallel lateral margins; fifth legs projecting at posterolateral angles. Abdomen and caudal rami as in preceding stage. Body length: 2.73 mm (2.66–2.81 mm) (*n* = 5).

**Figure 9.**
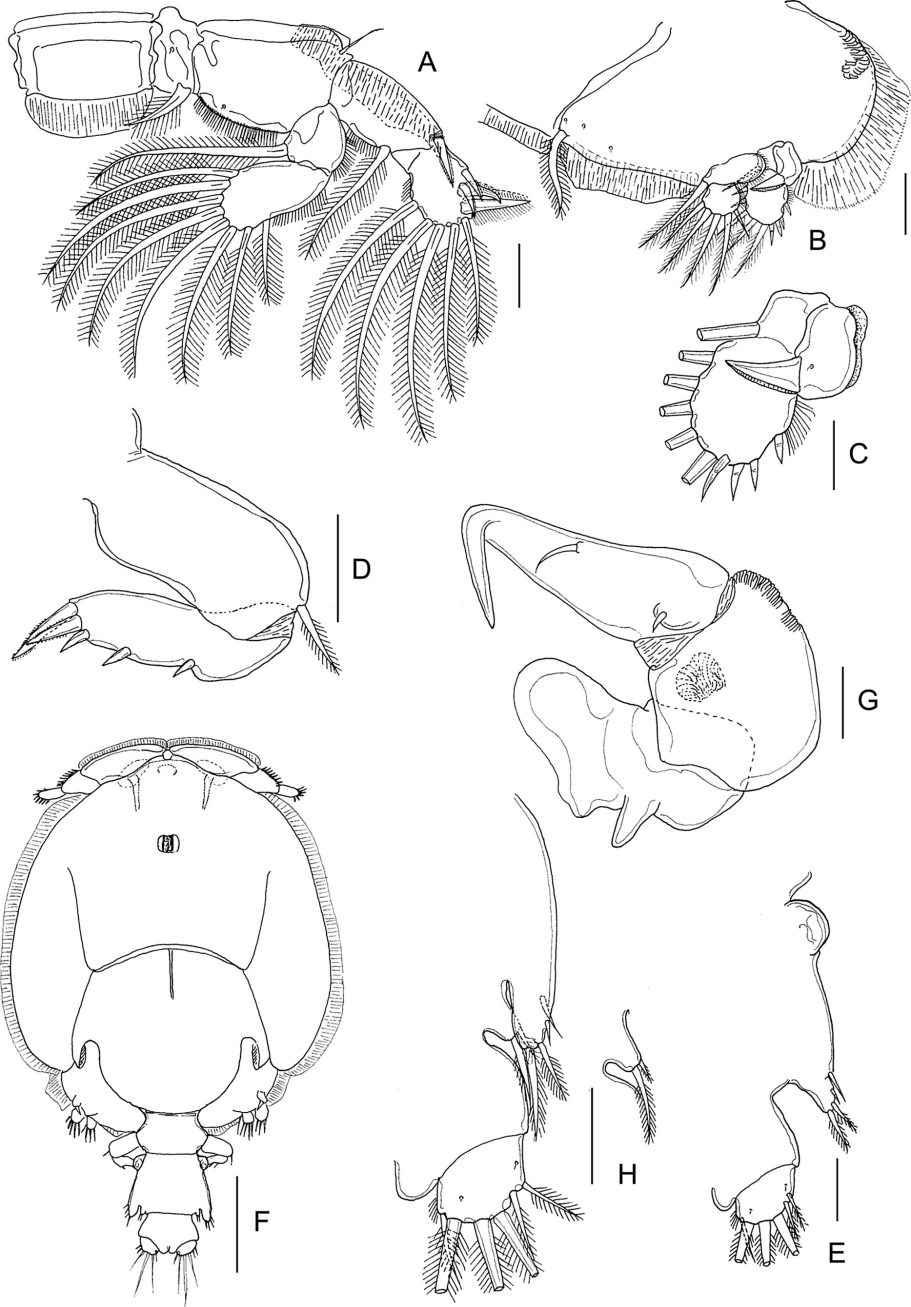
*Lepeophtheirus elegans* Gusev, 1951. Pre-adult I. Female (A–E): A, leg 2; B, leg 3; C, exopod of leg 3; D, leg 4; E, legs 5 and 6. Male (F–H): F, habitus, dorsal; G, antenna; H, legs 5 and 6. Scale bars: A–E, G, H = 0.1 mm; F = 0.4 mm.

Antennule (Figure 8B) proximal segment with array of 20 plumose setae, 18 on anteroventral surface and 2 on dorsal surface; distal segment unchanged. Antenna (Figure 8C) three-segmented as in adult; basal segment short, with well-developed posterior process; second segment robust, with reniform adhesion pad on dorsal surface near base of terminal segment and with corrugated surface anteroventrally; terminal claw strongly curved, armed with seta near base and longer seta at midlength. Postantennary process (Figure 8F) curved and tapering distally, with two multisensillate papillae on basal part and one on adjacent cephalothoracic surface, as in adult. Rounded process present on adjacent ventral cephalothoracic surface, medial to base. Mandible (Figure 8D) unchanged. Maxillule (Figure 8E) of typical adult form, with anterior sclerite, small papilla bearing three unequal setae, and bifid posterior process; inner tine relatively larger than in preceding stage. Maxilla (Figure 8G) of adult form; basal segment robust; distal segment elongate bearing flabellum at midlength, calamus and canna curved, claw-like, ornamented with minutely serrated membranous strips. Maxilliped (Figure 8H, I) as in preceding stage. Sternal furca (Figure 8J) well developed with broad box and divergent, slightly tapering tines.

Leg 1 (Figure 8K) of adult form; legs joined by slender interpodal bar (intercoxal sclerite); laterally directed exopod elongate, distinctly two-segmented; endopod vestigial (arrowed on Figure 8K), incompletely fused to basis, and bearing two minute vestigial elements at apex; sympod armed with pinnate seta on medial margin and slender outer seta; proximal exopodal segment with outer distal spine and ornamented with row of setules along medial margin, distal segment with three long pinnate setae on medial margin, spines 1–3 decreasing in size from outer to inner, each ornamented with row of spinules and each with pecten adjacent to origin on segment, seta 4 longer than spine 3 but shorter than segment (Figure 8L).

Leg 2 (Figure 9A) biramous, joined by broad intercoxal plate ornamented with marginal membrane along free posterior margin. Coxa short, with pinnate seta and single sensilla; basis with outer distal seta, inner margin ornamented with spinule row and single sensilla. Exopod two-segmented; anterior margin of proximal exopodal segment bearing marginal membrane reflexed back over dorsal surface of ramus; armed with outer spine and inner seta; distal segment with three short spines ornamented with marginal membrane, outer distal spine ornamented with membrane laterally and pinnules medially, and five pinnate setae. Endopod two-segmented; proximal segment shorter than distal, with long pinnate seta on medial margin and row of spinules along lateral margin, distal segment with eight pinnate setae, lateral margins of both segments fringed with spinules.

Leg 3 (Figure 9B) forming flattened plate closing posterior part of cephalothoracic sucker as in adult. Leg pair joined by plate-like, intercoxal sclerite bearing marginal membrane posteriorly. Protopodal part flattened, coxa bearing inner pinnate seta at junction with intercoxal plate, and outer plumose seta near base of exopod; sensilla located adjacent to inner coxal seta; ornamented with extensive membrane posteriorly, medial to endopod, and laterally, anterior to exopod; anterolateral angle of protopod produced into surface ridges. Both rami distinctly two-segmented; proximal segment of exopod (Figure 9C) with pinnate seta on medial margin and stout spine at outer distal angle reflexed over surface of ramus; distal segment with four short spines and five pinnate setae; proximal segment of endopod expanded laterally to close off space between rami, armed with pinnate seta on medial margin, distal segment with five long pinnate setae on medial. Setal formula is given as follows:

**Table:** 

	Coxa	Basis	Exopod	Endopod
Leg 1	0-0	1-1	I-0; III, I, 3	2 (vestigial)
Leg 2	0-1	1-0	I-1; III, I, 5	0-1; 8
Leg 3	(1-1)		I-1; III, I, 5	0-1; 5

Leg 4 (Figure 9D) uniramous, two-segmented; protopodal segment with single pinnate seta at outer distal angle; exopod unsegmented with two naked spiniform elements on lateral margin and three unequal elements on distal margin, two larger elements ornamented with fine spinules along margins.

Leg 5 (Figure 9E) represented by lobe at each posterolateral corner of genital complex, armed with four setae, distal two pinnate.

#### Pre-adult I, male (Figure 9F–H)

Sexual dimorphism expressed shape of genital complex, in the presence of leg 6 on genital complex and in fine ornamentation of antenna. Genital complex (Figure 9F) with lateral margins narrowing anteriorly, and bearing conspicuous paired leg 6 on posterior margin just medial to leg 5 (Figure 9H). Antenna (Figure 9G) as female, but slight differences in the extent of corrugations on surface of middle segment. Leg 6 (Figure 9H) consisting of lobate process on posteroventral surface of genital complex, just medial to leg 5; armed with two (one long and one short) pinnate setae. All other appendages similar to female. Body length: 2.27 mm (2.21–2.38 mm) (*n* = 6).

#### Pre-adult II, female (Figures 10, 11A–E)

**Figure 10.**
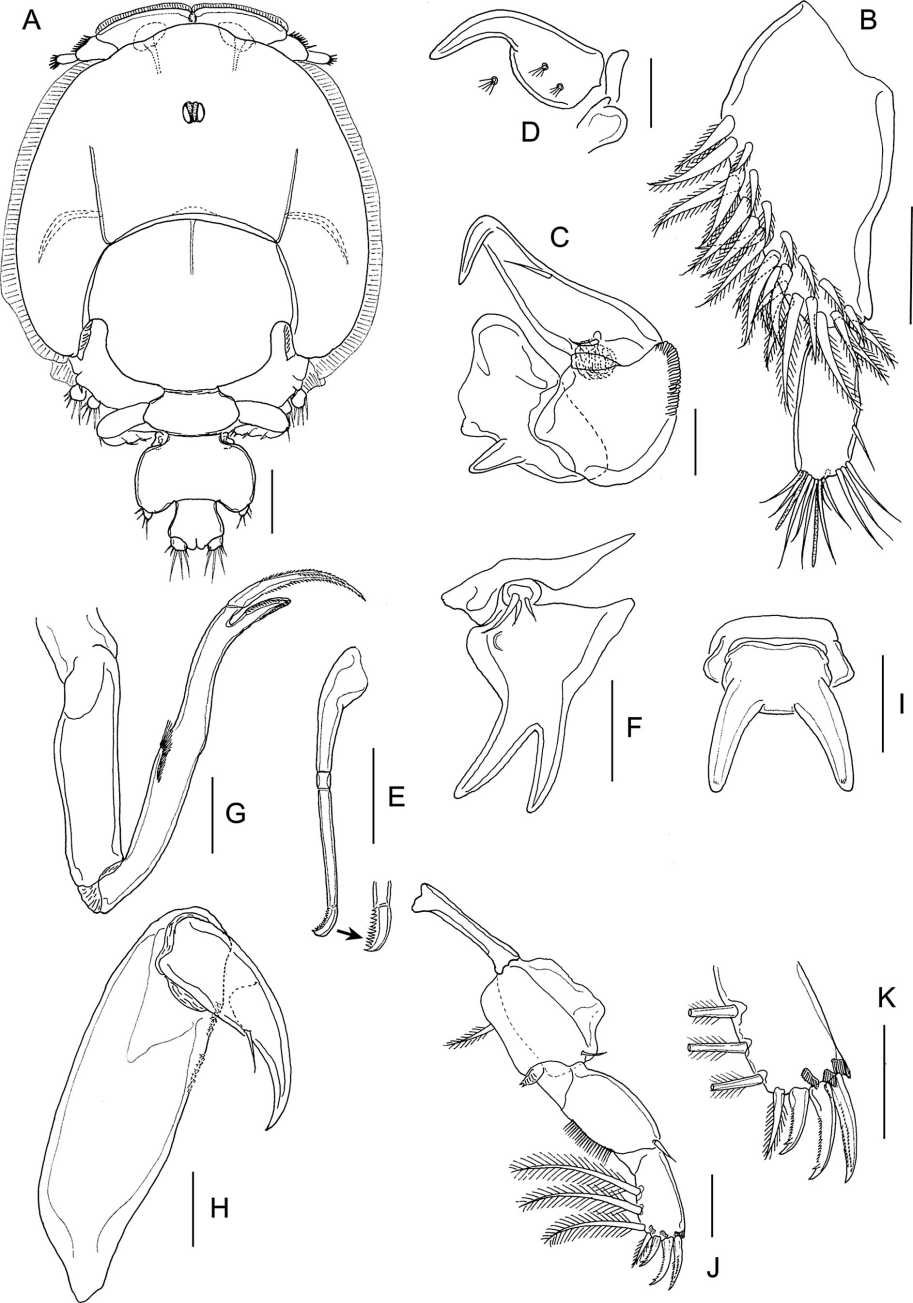
*Lepeophtheirus elegans* Gusev, 1951. Pre-adult II, female: A, habitus, dorsal; B, antennule; C, antenna; D, postantennary process; E, mandible; F, maxillule; G, maxilla; H, maxilliped; I, sternal furca; J, leg 1; K, tip of leg 1 exopod. Scale bars: A = 0.4 mm, B–K = 0.1 mm.

**Figure 11.**
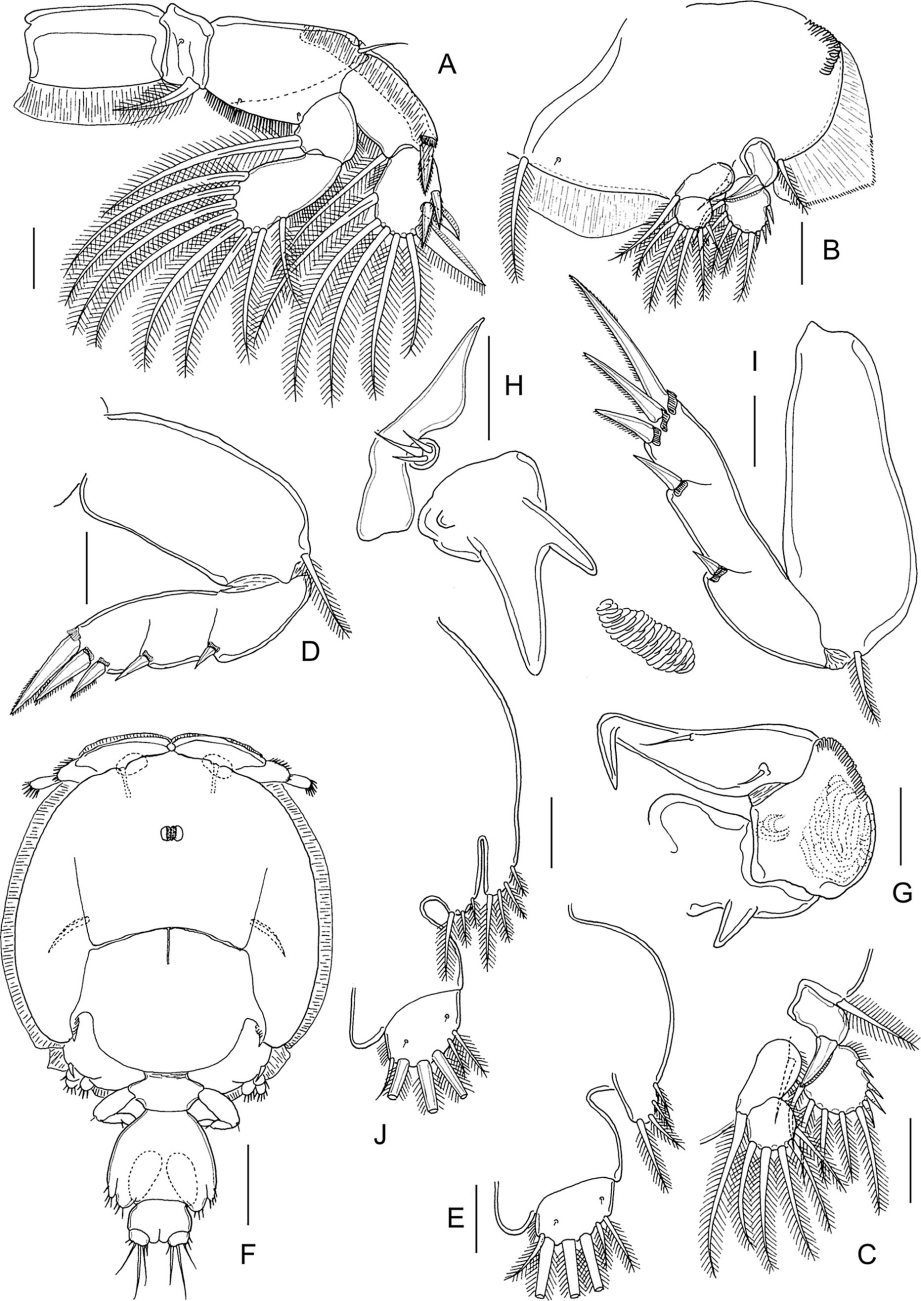
*Lepeophtheirus elegans* Gusev, 1951. Pre-adult II. Female (A–E): A, leg 2; B, leg 3; C, rami of leg 3; D, leg 4; E, leg 5 and caudal ramus. Male (F–J): F, habitus, dorsal; G, antenna; H, maxillule; I, leg 4; J, leg 5, leg 6 and caudal ramus. Scale bars: A–E, G–J = 0.1 mm; F = 0.4 mm.

General appearance of body (Figure 10A) as in preceding stage; cephalothorax about two times longer than post-cephalothoracic somites and caudal rami combined. Genital complex slightly produced to form weak rounded lobes at posterolateral corners; free abdomen and caudal ramus (Figure 11E) unchanged. Body length: 3.69 mm (3.57–3.78 mm) (*n* = 5).

Antennule (Figure 10B), proximal segment with 25 plumose setae along anteroventral margin and two setae dorsal to anterior margin; distal segment unchanged. Antenna (Figure 10C) unchanged. Postantennary process (Figure 10D) unchanged. Mandible (Figure 10E) unchanged. Maxillule (Figure 10F) as in preceding stage except with inner tine on posterior process almost as long as outer. Maxilla (Figure 10G) with distal segment more slender than in preceding stage. Maxilliped (Figure 10H) as in preceding stage. Sternal furca (Figure 10I) unchanged.

Leg 1 (Figure 10J–K) unchanged. Leg 2 (Figure 11A) with two-segmented rami as in preceding stage; distal segments of both rami relatively longer than in proceding stage. Leg 3 (Figure 11B, C) as preceding stage. Setal formula is given as follows:

**Table:** 

	Coxa	Basis	Exopod	Endopod
Leg 1	0-0	1-1	I-0; III,I,3	2 (vestigial)
Leg 2	0-1	1-0	I-1; III,I,5	0-1; 8
Leg 3	(1-1)		I-1; III,I,5	0-1; 5

Leg 4 (Figure 11D) exopod with two partial suture lines, each marking plane of incipient subdivision; lateral margin with two spines, one per incipient segment; three unequal distal spines armed with fine spinules; all spines with pecten at base. Leg 5 (Figure 11E) represented by lobate process on ventrolateral surface of genital complex, with four pinnate setae.

#### Pre-adult II, male (Figure 11F–J)

General appearance (Figure 11F) as in female, but differing in the shape of genital complex and abdomen. Genital complex less broad than that of female, narrow anteriorly and wider posteriorly; legs 5 and 6 located at posterolateral corners. Free abdominal somite shorter than in female. Appendages similar to those of female, except for antenna, maxillule, and legs 4–6. Antenna (Figure 11G) showing more extensive corrugations on surface of middle segment. Maxillule (Figure 11H) with corrugated adhesion pad present on adjacent ventral cephalothoracic surface. Leg 4 (Figure 11I) with spines better developed than in female. Leg 5 (Figure 11J) consisting of conical lobe on posterolateral margin of genital complex, armed with four pinnate setae. Leg 6 (Figure 11J) located just medial to leg 5, consisting of conical lobe armed with three pinnate setae. Body length: 2.61 mm (2.45–2.74 mm) (*n* = 5).

#### Adult female (Figures 12 and 13)

**Figure 12.**
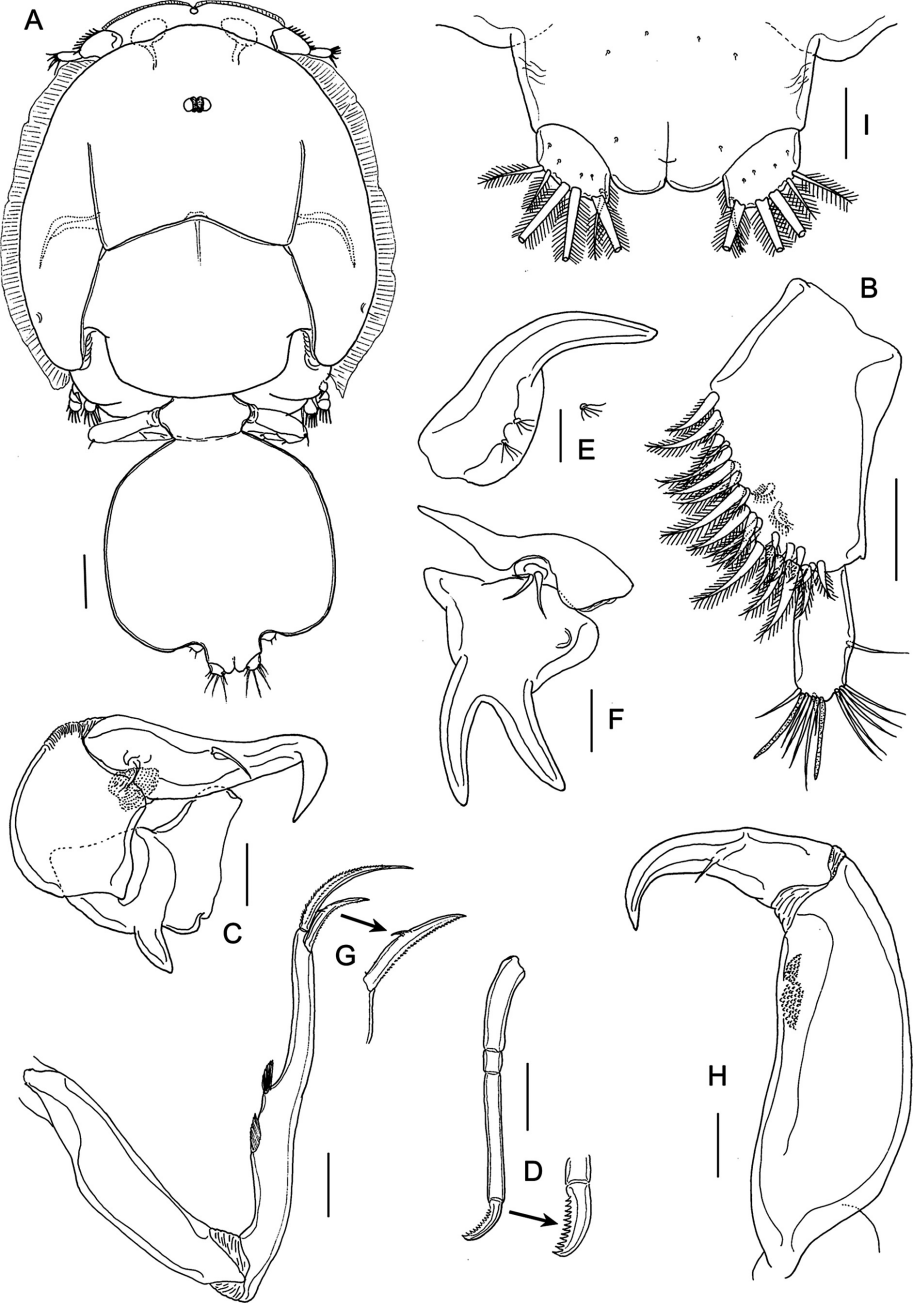
*Lepeophtheirus elegans* Gusev, 1951. Adult female: A, habitus, dorsal; B, antennule; C, antenna; D, mandible; E, postantennary process; F, maxillule; G, maxilla; H, maxilliped; I, abdomen, ventral. Scale bars: A = 0.4 mm; B–I = 0.1 mm.

Body (Figure 12A) with typical caligiform cephalothorax as in preceding stage, short free fourth pedigerous somite, large genital complex, and single free abdominal somite. Cephalothorax unchanged from preceding stage. Genital complex enlarged, just wider than long and with rounded posterolateral corners lacking processes. Abdomen and genital complex not separated by defined articulation. Surface of abdomen and caudal rami ornamented with symmetrical pattern of sensilla (Figure 12I). Body length: 4.79 mm (4.60–5.05 mm) (*n* = 10).

**Figure 13.**
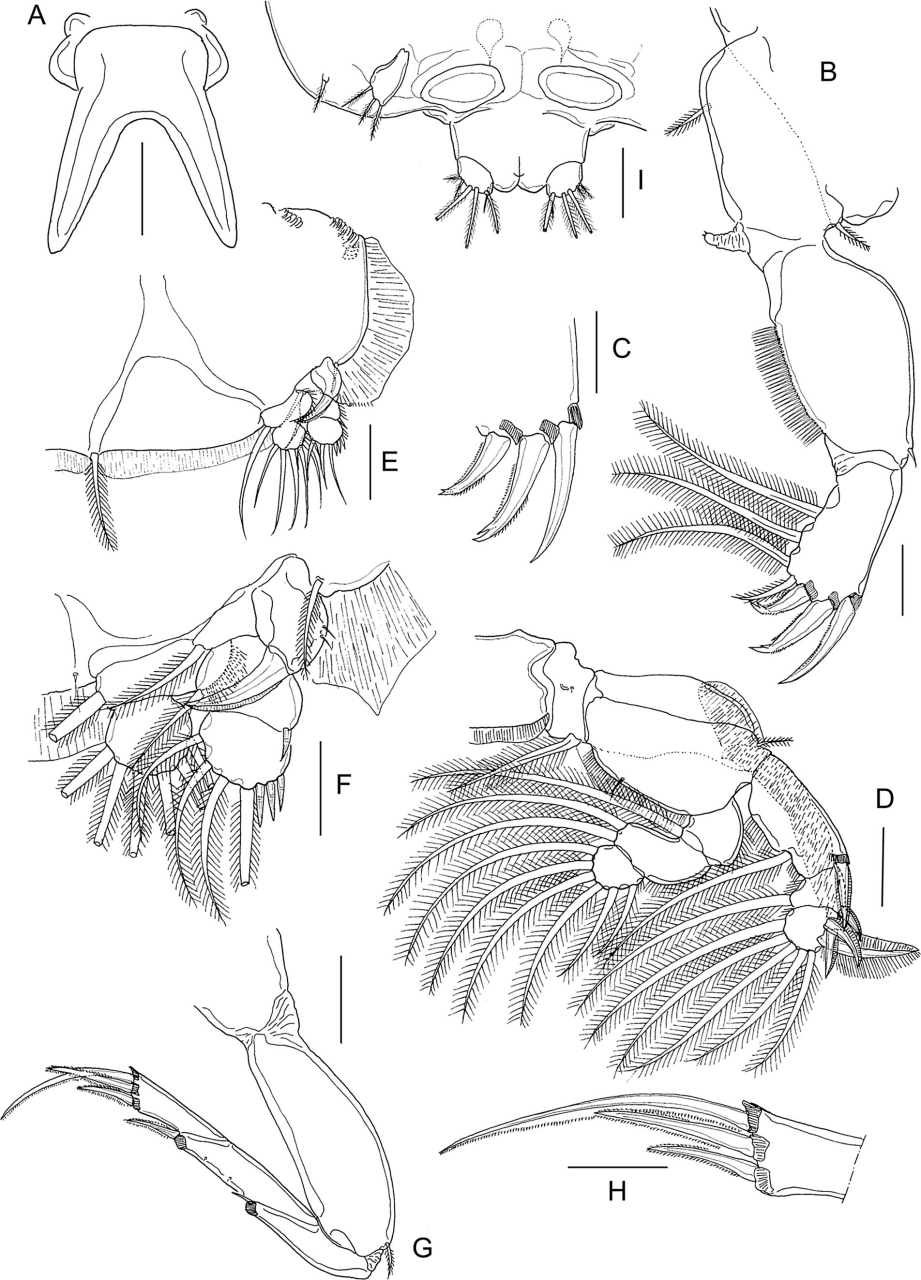
*Lepeophtheirus elegans* Gusev, 1951. Adult female: A, sternal furca; B, leg 1; C, detail of spines on tip of leg 1 exopod; D, leg 2; E, leg 3; F, endopod and exopod of leg 3; G, leg 4; H, tip of leg 4; I, abdomen, leg 5 and genital apertures with spermatophores attached. Scale bars: A, H = 0.1 mm; B–G, I = 0.2 mm.

Antennule two-segmented (Figure 12B); large proximal segment with 25 plumose setae along anteroventral margin and two setae located dorsally; distal segment bearing 14 elements (12 setae plus 2 aesthetascs) including isolated seta on posterior margin. Antenna (Figure 12C) with posteriorly directed spatulate process on proximal segment; middle segment subrectangular, with dorsal adhesion pad and with corrugations on anterodorsal surface; terminal part forming strong, recurved claw armed with slender seta midway along anterior margin and with smaller seta on raised knob located proximally. Postantennary process (Figure 12E) weakly curved; ornamented with two multisensillate papillae on basal part and with similar papilla on adjacent ventral cephalothoracic surface. Mandible (Figure 12D) of typical stylet-like structure with 12 marginal teeth subterminally. Maxillule (Figure 12F) comprising anterior sclerite, small papilla bearing three unequal setae, and bifid posterior process with both tines of similar size and slightly outwardly curved. Maxilla two-segmented (Figure 12G), comprising syncoxa and elongate basis: basis bearing subdivided flabellum on anterior margin, armed with two unequal claw-like elements (calamus and canna) distally. Calamus nearly twice as long as canna, both ornamented with strips of serrated membrane; canna with denticle. Maxilliped subchelate (Figure 12H); proximal protopodal segment robust, unarmed, medial surface ornamented with rugose patches distally; distal subchela with trace of suture separating short apical claw from proximal segmental part; armed with seta at midlength. Sternal furca with divergent, tapering tines (Figure 13A).

Leg 1 (Figure 13B); legs joined by slender interpodal bar; sympod armed with pinnate seta on medial margin and plumose seta on outer margin; endopod vestigial, incompletely fused to basis, with two minute vestigial elements at apex: laterally directed exopod elongate, distinctly two-segmented, proximal exopodal segment swollen near midlength, armed with small outer distal spine and ornamented with row of setules along posterior margin; distal segment with three large pinnate setae on medial margin, spines 1–3 decreasing in size from outer to inner, each ornamented with row spinules and each with pecten adjacent to origin on segment, spines 2 and 3 each with short accessory process; seta 4 longer than spine 3 but shorter than segment and shorter than spine 2 (Figure 13C).

Leg 2 (Figure 13D) biramous, with flattened protopod and three-segmented rami; coxae joined by plate-like, intercoxal sclerite bearing marginal membrane posteriorly. Coxa armed with pinnate seta and surface sensilla. Basis armed with outer plumose seta; ornamented with marginal membrane posteriorly, and membrane anteriorly, reflexed over dorsal surface of segment, long surface sensilla present near posterior margin. First exopodal segment with inner pinnate seta and with large outer spine ornamented with bilateral membranes, aligned with longitudinal axis of ramus; second segment with similar outer spine and inner seta; third segment with two outer spines each with bilateral membrane (proximal spine overlying base of distal spine), apical spine with marginal membrane laterally and pinnules medially, and five inner pinnate setae. Endopodal segments 1 and 2 armed with 1 and 2 inner pinnate setae respectively; segment 3 with 6 pinnate setae; outer margins of all endopodal segments with fine setules.

Leg 3 pair (Figure 13E) forming flattened plate closing posterior part of cephalothoracic sucker; leg pair joined by intercoxal plate ornamented with marginal membrane posteriorly. Protopodal part bearing pinnate inner coxal seta at junction with intercoxal plate, and outer seta near base of exopod; sensilla located adjacent to inner coxal seta; protopod ornamented with membrane along posterior margin medial to endopod and along lateral margin anterior to exopod. Exopod three-segmented (Figure 13F); first segment with inner pinnate seta and large, curved, outer spine reflexed over surface of ramus; second segment with slender outer spine and inner pinnate seta; third with seven setal elements (three outer spiniform elements and four inner pinnate setae). Endopod two-segmented (Figure 13F); first endopodal segment with inner pinnate seta; second with six pinnate setae. Spine and seta formula is given as follows:

**Table:** 

	Coxa	Basis	Exopod	Endopod
Leg 1	0-0	1-1	I-0; III, I, 3	2 (vestigial)
Leg 2	0-1	1-0	I-1; I-1; III, I, 5	0-1; 0-2; 6
Leg 3	(1-1)		I-1; I-1; III, I, 4	0-1; 6

Fourth leg (Figure 13G) four-segmented, comprising robust protopodal segment and three-segmented exopod with exopodal segments separated by oblique articulations. Protopodal segment armed with outer distal seta. First exopodal segment with minute outer spine with pecten at base; second segment with outer spine with pecten at base; third exopodal segment armed with three unequal distal spines, each with pecten at base (Figure 13H); spines on second and third segments ornamented with bilateral rows of minute denticles, except longest spine with row only along outer margin.

Fifth leg located posteroventrally on genital complex, represented by outer protopodal seta originating on somite surface and small exopodal lobe bearing three setae (Figure 13I). Sixth leg represented by unarmed plate closing off genital opening.

#### Adult male (Figure 14)

**Figure 14.**
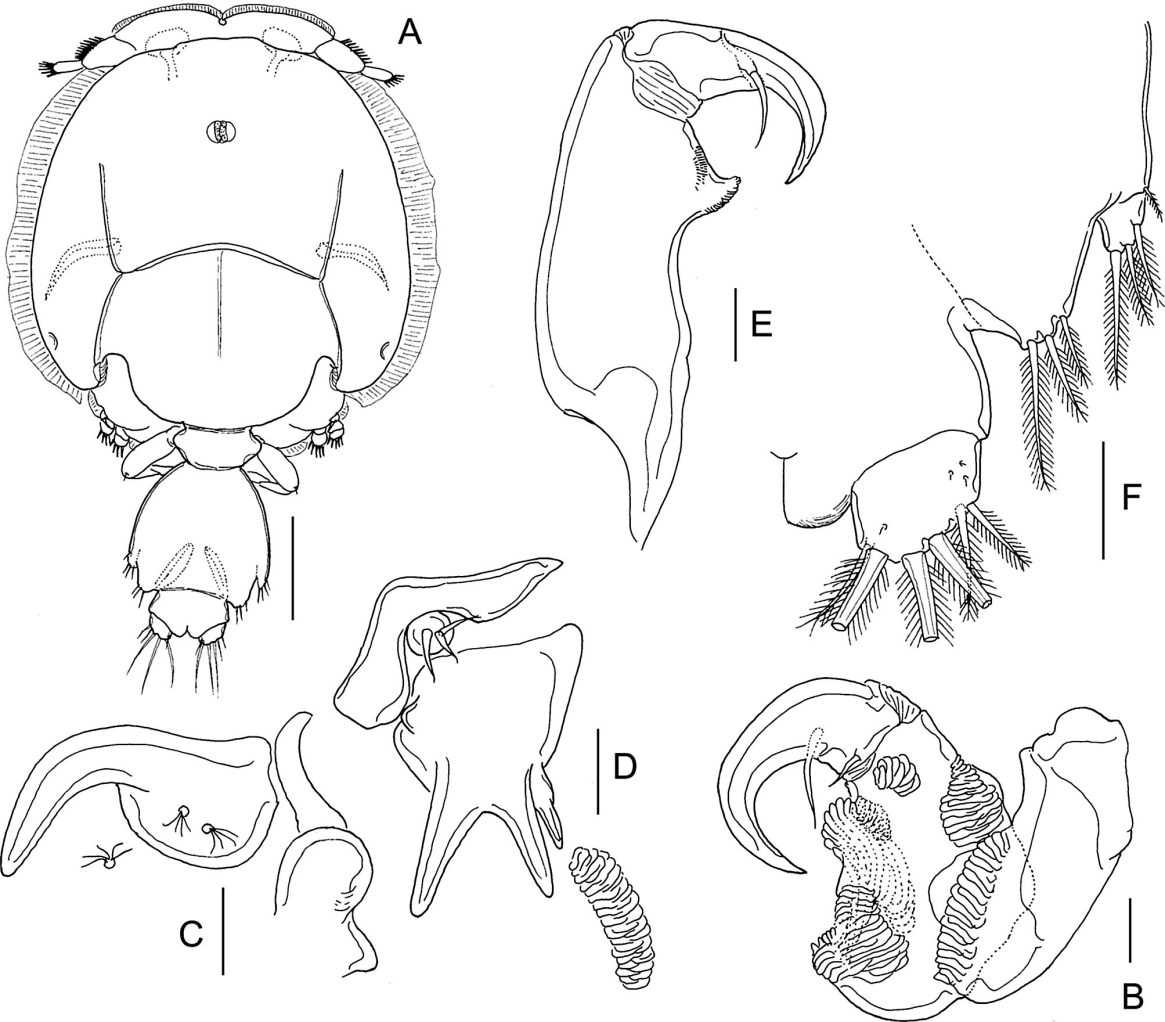
*Lepeophtheirus elegans* Gusev, 1951. Adult male: A, habitus, dorsal; B, antenna; C, postantennary process; D, maxillule; E, maxilliped; F, legs 5 and 6 and caudal ramus. Scale bars: A = 0.4 mm; B–E = 0.05 mm; F = 0.1 mm.

Cephalothorax as in female; genital complex relatively smaller than that of female, narrow anteriorly and widening slightly posteriorly; leg 5 located posteriorly on lateral margin of complex; leg 6 located at posterolateral corners. Free abdominal segment separated from abdomen by articulation (Figure 14A). Body length: 2.68 mm (2.54–2.94 mm) (*n* = 10).

Appendages similar to those of female, except for antenna, maxillule, maxilliped, and legs 5 and 6. Antenna (Figure 14B) modified, with corrugations on distal margin of proximal segment; middle segment with two discrete adhesion pads plus extensive corrugations over medial surface; distal segment forming strongly recurved hook bearing two setae proximally. Claw of postantennary process (Figure 14C) more strongly curved than in female. Maxillule (Figure 14D) with accessory (third) medial tine present on base of bifid posterior process: corrugated adhesion pad present on adjacent ventral cephalothoracic surface. Maxilliped with conspicuous myxal process located distally (Figure 14E), opposing tip of maxilliped claw. Leg 5 (Figure 14F) consisting of conical lobe located posteriorly on lateral margin of genital complex, armed with three pinnate setae and with protopodal seta adjacent to base. Leg 6 (Figure 14F) located just medial and posterior to leg 5, consisting of conical lobe armed with three pinnate setae.

## Discussion

The first segment of the antennule is armed with three setae in the infective copepodid stage of *L. elegans*, this increases to seven setae in chalimus I, 13 in chalimus II, 20 in pre-adult I, and 27 in pre-adult II. The number remains unchanged from pre-adult II to the adult (both have 27 setae in both sexes). This approximate pattern is shared with the two other species of *Lepeophtheirus* and with two of the species of *Caligus* (*C. epidemicus* Hewitt, 1971 [14] and *C. rotundigenitalis* Yü, 1933 [49]) listed by Ohtsuka *et al.* [35]. This pattern may also be shared with *Alebion lobatus* Cressey, 1970 [9], although our knowledge of the life cycle of this species is probably incomplete [3, 35]. Using the number of setae present on the proximal antennulary segment as a stage marker allows us to infer that the post-naupliar phase of the life cycle of *L. elegans* comprises only six stages. These are: copepodid, chalimus I, chalimus II, pre-adult I, pre-adult II, and the adult, but this clashes with the hitherto accepted pattern for *Lepeophtheirus* species which recognizes four chalimus stages as well as two pre-adults [4, 19, 28, 45].

In *L. elegans* the moult from any one of these stages to the next is marked by an increase in the number of setae on the proximal antennulary segment, except for the final moult from pre-adult II to adult. There is no setal addition here but the existence of a moult between these two stages is demonstrated unequivocally by the change in segmentation (from two to three-segmented) of both rami of leg 2 and of the exopod of leg 3 and by the appearance of prominent secondary sexual characters. In the male the final moult from pre-adult II to adult is marked by the sexually dimorphic transformation of the antenna, by the appearance of the third tine on the posterior process of the maxillule, and by the appearance of the myxal process on the protopod of the maxilliped. These secondary sexual characters appear at the final moult in the male. The existence of a moult separating pre-adult II and adult was confirmed by direct observation by Anstensruud [1] in a congener, *L. pectoralis*.

In this account we demonstrate the presence of just six post-naupliar stages in the life cycle of a *Lepeophtheirus* species. Six post-naupliar stages are also present in *Caligus* and *Pseudocaligus* although the nomenclature is different (see Discussion in Ref. [35]). In *Caligus* and *Pseudocaligus* the infective copepodid and the adult are separated by four distinct moult stages, termed chalimus I to chalimus IV. All four chalimus stages are attached to the host by a frontal filament which typically has new material added to it at each moult (e.g., [24]). There are no pre-adult stages in *Caligus* [36, 37]. In *L. elegans*, and we propose in all *Lepeophtheirus* species, the infective copepodid and the adult are also separated by four distinct moult stages, but these are termed chalimus I, chalimus II, pre-adult I, and pre-adult II. The two chalimus stages are permanently attached to their host, as in *Caligus* species, but the two pre-adult stages are distinguished by their ability to detach from the temporary frontal filament secreted during moulting and move over the surface of the host as observed by Anstensrud [1]. We conclude that all caligids have the same number of stages in the life cycle: two naupliar stages, the infective copepodid, a total of four chalimus and/or pre-adult stages, and the adult. This confirms the suggestion made by Oktsuka *et al.* [35] that in *Lepeophtheirus* the stages formerly treated as “chalimus I and II” represent a single stage (here termed chalimus I) and that the stages formerly treated as “chalimus III and IV” represent a single stage (here termed chalimus II). This is a profound change to the orthodoxy, with significant implications for the aquaculture industry, given that lice monitoring protocols include counts of chalimus stages and use temperature to predict when they will moult into the more pathogenic, mobile pre-adults. Lice management strategies, such as the periodic use of moult-inhibitors, must be tailored to the precise life cycle of the parasite.

In *L. elegans* we observed variation in body length in both chalimus I and chalimus II. Sample sizes were small (10 measurements for chalimus I and 13 chalimus II) but the distributions of length measurements were bimodal in each case. Initially we interpreted these as representing early and late individual instars, with the size differences reflecting intramoult growth, however we now consider it more likely that the two size morphs are males and females. In caligid life cycles sexual dimorphism has most commonly been reported as commencing at chalimus III in *Caligus* species and at pre-adult I (the equivalent stage) in *Lepeophtheirus* species [4, 24, 28–30, 36]. At this stage – the fourth post-naupliar stage of both genera – sexually dimorphic differences in limbs are first expressed and the sexes are readily recognisable. Prior to this stage, minor sexually dimorphic differences have sometimes been noted in size and shape in the preceding stage of some caligid species (e.g., [16, 39] but not in others (e.g., [4, 28, 29, 36]). Sexual dimorphism was noted in the “fourth chalimus” of *Caligus epidemicus* [29] but this stage is equivalent to chalimus II as used here because there were misinterpretations in the identification of the number of stages, as already noted by Ho & Lin [16] (see also discussion in Ohtsuka *et al*. [35]).

**Figure 15.**
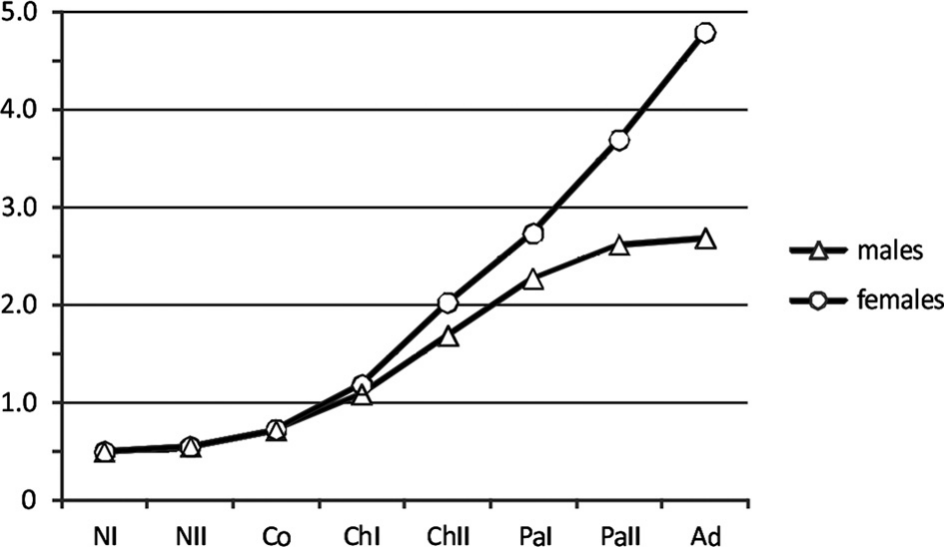
Mean body lengths (mm) of life cycle stages of *Lepeophtheirus elegans* Gusev, 1951, showing onset of sexual size dimorphism in chalimus I stage. NI, NII, nauplius I, II; Co, copepodid; ChI, ChII, chalimus I, II; PaI, PaII, pre-adult I, II; Ad, adult.

We consider it likely that the larger measured specimens of chalimus I are female, and the smaller ones are male; similarly with chalimus II, the larger specimens are probably female and smaller are males. In a species such as *L. elegans* where there is a very marked sexual size difference between adults (mean length of adult males 2.68 mm, mean length of adult females 4.79 mm), it is not surprising to find that the divergence in size between the sexes is first expressed early in development (Figure 15). In contrast, in a caligid such as *Caligus punctatus* Shiino, 1955 [41] where the adults are very similar in size (mean length of adult males 2.81 mm, mean length of adult females 2.96 mm), it is only when the limbs begin to express sexual dimorphism at chalimus III that the sexes can be distinguished [24]. We conclude that the failure to recognize sexual dimorphism in body size in the two earliest chalimus stages is the reason why the confusion had arisen concerning the number of post-naupliar stages in *Lepeophtheirus* species.

Ohtsuka *et al.* [35] identified two basic patterns for antennulary setal development in caligids: looking at the number of setae on the proximal antennulary segment through the post-naupliar stages they found a “3 : 7 : 13 : 20 : 27 : 27” pattern in *Lepeophtheirus* species, two *Caligus* species, and possibly in *Alebion* Krøyer, 1863 [27], and contrasted it with a “3 : 3 : 7 : 18 : 27 : 27” pattern found in other *Caligus* species and in *Pseudocaligus fugu*. These patterns were approximate, given some variation in original setal counts between species, but Ohtsuka *et al.* [35] considered that several setal counts required verification as some of them were clearly erroneous. The antennulary setation patterns of the chalimus stages of all caligid species for which the life cycle is known were subsequently tabulated by Madinabeitia & Nagasawa [30]: their table did not include the copepodid stage (three setae) but demonstrates substantial apparent variability within the two general patterns identified by Ohtsuka *et al.* [35]. However, their table contains errors, for example the setation pattern for *Caligus rotundigenitalis* is given as (3), 3, 7, 22, 29, 27–29 by Madinabeitia & Nagasawa [30] citing Ho & Lin [16] as the source. However, inspection of the figures in Ho & Lin [16] shows the following pattern: 3, 7, 11, 20, 27, 27 (as given in Ref. [35]). These errors are important since the pattern shown in Ho and Lin’s figures of *C. rotundigenitalis* can be attributed to the *Lepeophtheirus*-type rather than to the pattern found in the majority of *Caligus* species. No doubt variation exists, as in all biological systems, but we consider that antennulary setation is likely to be highly conserved within the family Caligidae, as in other copepods [7], and that much of the variability apparent from comparisons of old descriptions is erroneous.

Given the basal position of *Alebion* in the caligid phylogenetic tree relative to both *Caligus* and *Lepeophtheirus* [8], it is possible that the “3 : 7 : 13 : 20 : 27 : 27” pattern is plesiomorphic for the Caligidae. This would lead to the further inference that the “3 : 3 : 7 : 18 : 27 : 27” pattern, as found in most other *Caligus* species and in *Pseudocaligus fugu* [30, 35], is derived. This could be interpreted as further evidence questioning the validity of *Pseudocaligus* as a genus. Kabata [20, 21] was not convinced that *Pseudocaligus* should be retained as a separate taxon, pointing out that there were no supporting characters other than the reduction of the fourth legs. In fact, Kabata [22: 170] was “inclined to think” that *Pseudocaligus* should be synonymized with its parent genus, *Caligus*. On the basis of molecular evidence Øines and Schram [34] also concluded that *Pseudocaligus brevipedis* (Bassett Smith, 1896 [2]) belonged within the genus *Caligus*. A recent morphology-based phylogenetic analysis [16] linked *Pseudocaligus* and *Pseudolepeophtheirus* Markevich, 1940 [31] together in a clade characterized by shared reduction in the fourth leg, but we consider that there is always a danger of spurious relationships emerging from analyses of parasitic taxa where secondary reduction is common. A subsequent review by Dojiri & Ho [10] concluded that *Pseudocaligus* should be treated as a junior synonym of *Caligus*.

Now that we have identified a common life cycle pattern for all caligids, the evidence of setal counts suggests the existence of a widespread setal development pattern common to *Alebion*, *Lepeophtheirus*, and at least some *Caligus* species. Our interpretation of this pattern as plesiomorphic leads us to infer that the other pattern shared by most *Caligus* species and by *Pseudocaligus fugu* is derived. The shared possession of a derived developmental pattern provides further evidence supporting the inference that *Pseudocaligus* originates from within the genus *Caligus*, and the proposal that *Pseudocaligus* should be relegated to synonymy with *Caligus* [10].
